# Knowledge sharing to support long‐term condition self‐management—Patient and health‐care professional perspectives

**DOI:** 10.1111/hex.13209

**Published:** 2021-02-06

**Authors:** Sarah Brand, Stephen Timmons

**Affiliations:** ^1^ Nottingham University Business School Nottingham UK

**Keywords:** expertise, health‐care professionals, knowledge sharing, long‐term conditions, patients, self‐management

## Abstract

**Background:**

Increased self‐management is a suggested solution to the burden on health‐care services of long‐term conditions (LTCs). This requires effective sharing of knowledge between health‐care professionals and patients, and is an underexplored area.

**Objective:**

To understand how patients and health‐care professionals (HCPs) share and utilize knowledge in the social context of health‐care interactions within long‐term condition management.

**Methods:**

Thematic analysis of 93 hours of observations of health‐care interactions and 33 semi‐structured interviews involving patients, carers and HCPs.

**Results:**

3 themes were identified: normative social roles, differing professional roles and the value of knowledge. Knowledge sharing was a complex process heavily influenced by social and cultural norms within the health‐care context. Not all knowledge was easily shared within routine health‐care interactions.

**Discussion:**

The social context in which health‐care is practised influences what knowledge is shared and how this is achieved. It favours sharing of clinical knowledge from HCPs to patients and disadvantages patients in their ability to share their unique knowledge based on lived experience of illness. The opportunities for patients to be supported in their knowledge, skills and confidence within routine health‐care interactions are limited.

**Conclusion:**

Both patients and HCPs need support to recognize the characteristics of the social context of health care and their understandings of their roles within this in order for them to move beyond accepted behaviours to develop more effective partnership working.

**Patient or Public Contribution:**

Patients were involved in initial design of the study, particularly ethics of ethnographic observation.

## 1 BACKGROUND

In the UK, effective management of long‐term conditions (LTCs) is seen to be one of the greatest challenges facing health services.^1^ Individuals' health‐related behaviours strongly influence the prevalence and course of disease and illness, and the associated requirement for health‐care interventions.^2^, ^3^ One of the proposed solutions for the challenge posed by increasing prevalence of LTCs is to support patients to better manage their conditions themselves, thereby reducing uptake of health‐care services and progression of severity of disease.^2^, ^4^, ^5^ This requires both patients and health‐care professionals (HCPs) to share knowledge and utilize it in order for supportive self‐management to take place. This paper seeks to explore how knowledge sharing takes place within the context of LTC management by:

- Problematizing knowledge itself
- Taking an alternative perspective to view how knowledge is utilized by social actors within health‐care decision making
- Illuminating the influence of the social context in which knowledge sharing takes place

Self‐management is broadly understood to encompass actions taken by individuals to recognize, treat and manage their own health and is associated with improved health outcomes.^6^, ^7^ Self‐management programmes aim to build individuals' knowledge, skills and confidence both in their condition and the management activities associated with it.^8^, ^9^, ^10^, ^11^ Self‐management and the associated engagement of patients in their health care are associated with increased uptake and sustainability of healthy behaviours and improved clinical outcomes.^9^, ^10^, ^11^ The assumption is that knowledge can be 'given' to patients; the main issue being ensuring that they then use this knowledge to inform their behaviours.^8^, ^11^, ^12^ This assumption is contrary to much literature concerned with knowledge within health‐care contexts which highlight the difficulties of knowledge sharing associated with organizational barriers, resource constraints, multi‐professional working and the constant production of new knowledge.^13^, ^14^

There is also an assumption that knowledge sharing is primarily a unidirectional activity with HCPs 'transferring' knowledge to patients. This is in contrast not only to the theory associated with knowledge sharing ^15^, ^16^ but also to personalized care models in which the lived experience of illness by patients is purported to be equally valued.^17^ It has been suggested that an increased focus on self‐management introduces a new paradigm centred around the patient‐professional partnership ^9^ which necessarily involves multi‐direction knowledge sharing.

Shared decision making is implicit in the personalized care agenda.^17^ It has been shown to have benefits for patients, carers and HCPs, but also challenges in its practical implementation.^18^, ^19^, ^20^, ^21^ The shared decision‐making literature tends to focus on communication and process; the knowledge involved is often unconsidered. By foregrounding and problematizing knowledge itself, its influence on sharing between social actors can be explored.

Health literacy is a related concept which has been widely discussed ^22^, ^23^, ^24^ and relates to the ability of patients to obtain, understand and use information in order to make appropriate health‐care decisions.^25^ Lower health literacy has been associated with poorer health outcomes and poorer utilization of health services.^26^ This results in less appropriate use of health services overall—increased use of emergency services, reduced engagement with preventative care and reduced ability to self‐manage routine health needs.^26^ Rather than considering how patients are able to obtain and understand health‐care information, this paper explores how easy it is for patients and HCPs to share this knowledge as social actors within interactions. This alternative lens through which to view health‐care interactions allows elucidation of some of the cultural and social issues at play within the health‐care context.

There are a range of social factors which can make knowledge sharing complex.^27^, ^28^ These factors relate to how society is organized, such as the social constructs of patient, nurse and doctor along with their associated activities and expectations. Social actors sharing knowledge come from different worlds, occupy different social roles associated with differential status and communicate in unique ways.^29^, ^30^, ^31^ It is these social factors in relation to knowledge sharing which the current paper seeks to explore. This paper aimed to illuminate the social context and the cultural norms associated with it, in which knowledge sharing takes place. Cultural norms encompass the social behaviours accepted and perpetuated by society, and can be exemplified by the patient and HCP roles.^32^ This context and accepted norms associated with it can be so embedded as to be imperceptible to the social actors operating within it, but nevertheless imperative to acknowledge in order to understand the setting in which health care is enacted.

## 2 Methods

### 2.1 Design

A qualitative approach underpinned by ethnographic principles^33^, ^34^, ^35^ was utilized to explore the behaviours associated with, and perceptions of, patients, carers and HCPs in relation to knowledge sharing.

### 2.2 Participants

Participants were patients who were diagnosed with a LTC and their carers, along with the HCPs who formed their immediate care team. Inclusion and exclusion criteria are shown in box 1. Data were collected from two large teaching hospitals in the UK East Midlands. One site cared for patients with renal disease, and the other, for patients with bowel and liver disorders. These disorders were chosen as they were conditions which affected both males and females of a range of ages and are relatively under‐researched in relation to other LTCs such as diabetes. Data were collected in 15 months during late 2015 and 2016.

Box 1 **Participant inclusion and exclusion criteria**

**Table:** 

**Patient inclusion criteria** Aged 18 or over Capacity to give informed consent Receiving treatment for target long‐term condition at study site **Patient exclusion criteria** Unable to give informed consent Unable to communicate adequately in English to allow participation in an interview **HCP inclusion criteria** Provided informed consent Observed in interactions with patient participants **HCP exclusion criteria** Unwilling to provide informed consent for observation of interactions with patient participants

21 patients and 2 carers, and 24 HCPs took part in the study. A purposive sampling approach for patient participants was utilized.^33^ The main theme used within this sampling approach was knowledge and expertise. To self‐manage, patients must have some level of knowledge and expertise in the practices required to manage their conditions. In addition, relationships with primary HCP were considered as it was conjectured that the character of these relationships may have an influence on how knowledge was shared as a socially situated activity. Carers were recruited to the study if they attended routine health‐care appointments with a patient, and their participation was therefore driven by the patient participants. Sampling considerations and demographics are shown in Table 1.

**TABLE 1:** Patient/carer demographics

Participant number	Gender	Ethnicity	Age	Rationale for selection
PN001	Male	White British	26	Extensive family history of similar conditions
PN002	Female	White British	30	Recently changed allocated consultant in order to get second opinion on treatment options
PN003	Male	White British	28	Initially treated in paediatric service, recently moved to adult service and requested change in allocated consultant
PN004	Female	White British	45	Newly diagnosed
PN005	Male	White British	52	Contracted viral illness as a result of receiving contaminated blood products as a child, politically active in patient support groups
PN006	Male	White British	55	Stigmatized viral illnesses, works within health services management
PN007	Male	White British	57	Multiple co‐morbidities, diagnosed for considerable period of time
PN008	Female	White British	53	Long‐term relationship with consultant who recently retired, now under new consultant
PN009	Female	White British	43	Impending change of current treatment
PN010	Male	White British	70	Long history of illness and co‐morbidities, very active in patient groups associated with condition, politically active
PL001	Male	White British	53	Selected by staff as 'expert patient' and participates in patient information days
PL002	Female	Mixed race	49	Selected by staff as 'expert patient' and participates in patient information days, current treatment likely to be altered fundamentally as condition deteriorating
PL003	Female	White British	74	Selected by staff as 'expert patient' and participates in patient information days, current treatment likely to be altered fundamentally as condition deteriorating
PL004	Male	Asian	63	Has instituted himself as an 'expert patient' although this is not recognized by staff, active in information giving to patients both within the service and externally
PL005	Female	White British	48	Treated in 'satellite unit' away from main hospital site, likely to require more intensive treatment as condition deteriorating. Considering transplantation which requires co‐operation of family members as potential donors
PL006	Male	White British	79	Likely to require more intensive treatment as condition deteriorating
PL007	Male	White British	61	Carries out intensive treatment independently at home
PL008	Female	White British	35	Daughter of PL007
PL009	Female	White British	71	Diagnosed for considerable period of time, active in fundraising and community patient support groups
PL010	Female	White British	61	Carries out home treatment, identified 'expert patient', treated out of main centre, multiple co‐morbidities
PL011	Female	White British	54	Carries out home treatment which is failing, impending change to treatments
PL012	Female	Indian	47	Active in patient groups, intensive treatment carried out regularly in hospital, multiple co‐morbidities, current treatment failing
PL013	Female	White British	61	Wife of PL007, involved in home treatment

HCP participants were those who were involved in the care of the patient participants and who were observed interacting with them. HCP participant characteristics are shown in Table 2. Demographic data were not collected on HCP participants.

**TABLE 2:** HCP characteristics

Participant number	Gender	Professional role	Rationale for interview selection
SN001	Female	Consultant	Oversight of entirely nurse‐led service
SN002	Female	Specialist nurse	Involved in nurse‐led service
SN003	Male	Consultant surgeon	NOT INTERVIEWED
SN004	Male	Consultant	*Refused interview*
SN005	Female	Consultant	Observed with multiple patients
SN006	Female	Specialist nurse	Observed with multiple patients
SN007	Female	Consultant	Participant in joint clinic
SN008	Male	Consultant	Not interviewed
SN009	Male	Dietician	Not interviewed
SN010	Female	Specialist nurse	Not interviewed
SN011	Male	Consultant	Not interviewed
SN012	Female	Specialist nurse	Involved in nurse‐led telephone advice service
SL001	Female	Specialist nurse	Not interviewed
SL002	Female	Specialist nurse	Investigation of multi‐disciplinary team
SL003	Male	Consultant	Observed with multiple patients
SL004	Male	Consultant	Observed with multiple patients, new to department
SL005	Female	Specialist nurse	Not interviewed
SL006	Male	Consultant	Not interviewed
SL007	Male	Consultant	Observed with multiple patients, oversight of home therapy patients
SL008	Female	Dietician	Not interviewed
SL009	Male	Consultant	Works outside of main treatment centre
SL010	Female	Dietician	Explore different professions perspective
SL011	Male	Consultant	Not interviewed
SL012	Female	Specialist nurse	Not interviewed

### 2.3 Ethical considerations

All participants were recruited to the study in compliance with ICH‐GCP requirements. This, and the process of ethical approval, ensured that processes were in place to protect participants from potential harm. In addition, all data were protected in line with General Data Protection Regulations and information governance requirements of both the academic sponsor of the study and the health‐care environment were chosen as study sites. None of the participants were known to the researcher prior to their participation. Initial general observation was undertaken within public areas such as outpatient clinics or patient information sessions. The aim of this was to allow the researcher to familiarize themselves with the general environment, key actors and processes in order to determine how more focused observation should be undertaken, and with whom. The researcher was identifiable and information regarding the conduct of a research study was provided to individuals located in the area. During general observation, the researcher conversed with patients who were in the environment and if appropriate discussed further participation in the research study which would involve observation of confidential consultations and interviews. Written information was provided to potential participants at this stage. Other potential participants were approached by their usual care provider and provided with written study information. Written informed consent was obtained prior to any further observation or interview. Four potential participants who were approached declined to participate.

East Midlands—Nottingham 1 NHS Research Ethics committee granted ethical approval (Ref 15/EM/0442).

### 2.4 Data collection

Data collection involved observations and ethnographic interviews. Initial general observations were undertaken of the environment, as detailed above, in order for the researcher to gain familiarity with the wider context in which the study was set, the key actors within it, and the specific environments in which further observation might be undertaken with participants. Subsequently, once patient participants had been selected and consented, observations took place of routine health‐care consultations, multi‐disciplinary meetings, patient‐led events and patient information sessions both in the hospital and at other locations such as patients' homes. Observations were patient participant‐led in that the schedule followed the patients' schedule of contact with the HCPs who were involved in their care. Attempts were made to observe all routine contacts patient participants had with specialist hospital services. 144 episodes of observation were undertaken covering 93 hours. Field notes were kept contemporaneously with observation, as well as a reflective diary which the researcher maintained after and between observations.

In addition, 33 interviews were undertaken with patients, carers and HCPs. All patients and carers were interviewed save for one patient who became too unwell, and selected HCPs were interviewed based on how much contribution they had made to the observational data as shown in Table 2. The interviews were semi‐structured, and whilst topics for interviews were common to both patients and HCPs, the questions asked varied between the two groups. A topic guide was developed by the researcher who also conducted the interviews. Examples of questions are shown in Table 3. Interviews were participant‐led, so the topic guide was used as an indicative tool, but the course of the interview was contingent on the participant. Interviews lasted between 30 minutes and 1 hour 37 minutes were carried out in the location of the participant's choice and involved the researcher and interviewee only. All interviews were recorded and transcribed verbatim. The transcripts, along with the field notes maintained during the observations, became the data for the analysis.

**TABLE 3:** Examples of interview questions

Areas for exploration	Interview questions (patients)	Interview questions (staff)
Where, when & why is knowledge shared?	1.1. Do you think that you know enough about your condition to enable you to make decisions about how to manage it? 1.2. Where do you get most of your information from? 1.3. If you felt that you needed more information, where would you look? Who would you ask? 1.4. How do you know when you need more information about something? 1.5. How do you know when you have enough information? 1.6. Do you think having more information is always a good thing?	1.7. How do you assess patient knowledge of their condition? 1.8. What information or knowledge do you feel the patient can contribute to illness management? 1.9. Do you think patients have increasing knowledge? 1.10. Is this always a good thing? 1.11. How do you know when the patient wants more information? 1.12. How do you know when patients have enough information? Can you give me an example 1.13. Is some knowledge or information which patients have more useful than others? Can you give me an example?

Data collection was undertaken by a single researcher. Selected sites were unfamiliar to the researcher in order to minimize researcher biases. The researcher had no knowledge of either the patients or staff at either site. It was a requirement of ethical approval that the researcher disclosed their role as a researcher and professional background, which is a qualified nurse, to all participants. This undoubtedly influenced the data which were collected. This may have been beneficial in some respects as patients were very open with the researcher and assumed some level of knowledge about the practicalities of health care and their disease which enabled them to move directly to discussing more personal perspectives of their health‐care experiences. Efforts were taken to minimize influence however as the researcher was not in uniform at any time, and data collected in organizations in which they were unknown in their professional role. Ultimately, it was felt that the position of the researcher enriched the data rather than restricted it, but there is no doubt that it had an influence.

### 2.5 Data analysis

Data were managed using NVivo software. Thematic analysis took place concurrently with data collection, and initial analysis informed subsequent data collection using the constant comparison method.^36^ This is commensurate with the ethnographic approach where analysis is an ongoing iterative process with constant movement between the field, generated data and emerging ideas.^34^ The units of analysis were the two groups of patients and health‐care professionals. Analysis was inductive, with open coding generating numerous categories which then was followed by focused coding involving synthesis of initial codes into categories.^37^ From 159 initial codes, 13 broad categories were formulated. A more abstract review of the data was then undertaken, considering the larger narrative and its relation to current theoretical understanding of knowledge sharing.^38^ The constant comparative method ensured that existing data were constantly compared with new data, resulting in categories being elaborated or integrated within the process of analysis.^36^ The initial analysis and coding was undertaken by one researcher, but then reviewed with two other researchers who were part of the study team. Broader themes and subsequent findings were discussed with all three researchers.

Data saturation was not an aim within this study; rather an attempt was made to gain a breadth and depth of experience and perspective. The limits to data collection were mainly pragmatic in terms of the number of participants a single researcher could adequately follow simultaneously, and the need for data collection to be concluded in the timeframe required. Utilizing the constant comparison method did show, however, that there were increasingly fewer amendments to existing codes as a result of new data generated and that data could be explained using existing codes and therefore data collection was discontinued.

## 3 RESULTS

Reported here are three main themes emergent from the data which illuminated how knowledge sharing was achieved between patients and HCPs within routine interactions.

- Normative social roles which focus on how social actors' understandings of their identity and what is expected of them influences how they behave within interactions.
- How differing professional roles within the health‐care sphere influence what knowledge is shared.
- Different types of knowledge prevalent in health care and the value assigned to these by social actors.

### 3.1 Normative social roles

It was evident that both HCPs and patients had clear understandings of their identity within interactions and what behaviour was expected when occupying these roles. This influenced how they approached knowledge sharing specifically. HCPs saw knowledge sharing as a key element of their role, particularly in relation to ensuring that patients had the essential knowledge required to understand and manage their conditions. It was assumed that it was the HCP's role to provide knowledge to patients who would know very little about their health conditions.

Just sort of assume that they know nothing. **SL007 (Renal doctor) Interview**

The patient role was also associated with a responsibility for knowledge sharing, but this was primarily to allow HCPs to make diagnosis and treatment decisions.

I think I tell them everything…it's always better to tell them as much as you know, to help them diagnose you or treat you. **PN009 (Gastro patient) Interview**

It was apparent that part of the patient role was not only to share knowledge, but also to follow the advice of the HCP. This was not articulated, but was apparent in observed behaviours.

The patient catches sight of the dietician and says that she hopes that she won't have to see her whilst she's there. She says that she doesn't find the dietician very helpful… so she just carries on doing her own thing. She puts her eyes down and tries not to catch the eye of the dietician. **PL001 (Renal patient) Observation**

It would appear that openly disregarding HCPs' knowledge and advice was difficult for many patients and they understood that this violated social norms. In contrast, HCPs did not find any difficulty in disagreeing with knowledge offered by patients.

I can look into it and say oh, did you know that this you know, this isn't quite right. **SN002 (Gastro nurse) Interview**

It was clear that whilst both HCPs and patients felt that it was their role to share knowledge with the other party, it was only incumbent on patients to utilize the knowledge shared with them. HCPs could more easily choose not to utilize the knowledge offered to them by patients.

### 3.2 Differing professional roles

This responsibility to share knowledge belied the complexity of knowledge sharing in practice however. It appeared that knowledge sharing differed dependant on the professional role of the HCP involved in the interaction. This was not only related to the knowledge shared by the HCP (which might be assumed given the different core knowledge bases of different professions) but also to the knowledge which patients shared with HCPs. This appeared to be related to the perceived empathy of the HCP with the patient perspective and the likely response to sharing of particular knowledge, rather than the knowledge which patients felt that individual would need to fulfil their professional role. HCPs appeared to be aware of this and accepted it as an element of multi‐disciplinary teamworking.

So many patients will tell the IBD nurses, tell her [the consultant] I don't want to do it. I'm not going to do it. Or tell her I don't do it. **SN005 (Gastro doctor) Interview**

This was corroborated by nursing staff.

I think they're [patients] much more honest with the nurses than the doctors. **SN012 (Gastro nurse) Interview**

Nurses tended to be used as conduits through which 'difficult' knowledge could be shared and communicated to other professional groups, primarily medical staff. This relates to the difficulty highlighted previously which patients found in disagreeing with some HCPs' proffered knowledge and advice. The input of the multi‐disciplinary team in caring for patients with LTCs appears to offer opportunity for more comprehensive knowledge sharing to take place however, particularly from patients to the HCP team.

### 3.3 Value of knowledge

It could be seen that there were two main types of knowledge prevalent in the health‐care context—clinical biomedical knowledge, mainly accessed by HCPs through training and accreditation, and experiential knowledge, largely associated with patients' lived experience of illness. It would appear that clinical knowledge and experiential knowledge were valued differently within the health‐care context both by HCPs and patients. Routinely, clinical knowledge appeared to be much more highly valued than experiential knowledge.

I'm not medically trained…..and I don't have the knowledge of anatomy and physiology to a level that an expert would have. **PN008 (Gastro patient) Interview**

[A] patient that has a lot of information based on their own pure experience, that's not really…everyone's different really. **SL007 (Renal doctor) Interview**

The lower value of patients' experiential knowledge appeared to be perpetuated within routine health‐care consultations. Within health‐care consultations, patients' knowledge could be easily dismissed, being overridden by the clinical knowledge and decision‐making power of the HCP.

The doctor says that it is worth trying an additional medication. The patient says she feels that her body is just getting to the point where it is feeling better and stronger. She asks if she can start the additional immunosuppression later when her body has had time to feel stronger still.…The specialist nurse comes in and the doctor discusses starting the new treatment with the nurse….A plan appears to have been formulated despite the patient not clearly agreeing to it. **PN002 (Gastro Patient) Observation**

The patient tries to contribute her experiential knowledge of how she feels and this is ignored in favour of a clinically focused treatment plan. In fact, the knowledge which she contributes is not even acknowledged. The value of clinical knowledge and the HCP's decision‐making power overrides the contribution of experiential knowledge which the patient makes.

One of the reasons for this may be associated with the normative role of the patient highlighted previously. It appeared that the role of patient was not commensurate with contributing clinical knowledge to health‐care interactions—this was the remit of the HCP. Patients reported experiencing issues if they attempted to contribute clinical knowledge within routine consultations concerned with their own health.

Even if I've come to a conclusion – always a hugely tentative conclusion – because I know it's not my line of work…..but if I present that conclusion, my experience is that that kind of ends the, the whole conversation. **PN007 (Gastro Patient) Interview**

Therefore, not only did clinical and experiential knowledge have different value within clinical consultations, but each type of knowledge was also closely associated with normative social roles; clinical knowledge with HCPs and experiential knowledge with patients. Patients attempting to contribute clinical knowledge to interactions was problematic. These normative social roles were perpetuated through routine practices of both HCPs and patients in order to ensure the smooth course of interactions.

Sort of respecting their [HCPs] position I suppose. Then you get the best out of…as a patient you are far more likely to get more out of that individual if you take that approach than if you take an approach that you know more. **PN006 (Gastro Patient) Interview**

Knowledge sharing was underpinned by normative social roles—how knowledge was valued, what knowledge could be shared, when and how— and itself perpetuated those roles within the practices seen in interactions—complying with the role expectations. Whilst patients recognized that it was their responsibility to share knowledge with HCPs, on deeper exploration, it is clear that not all knowledge to which the patient had access was shared, as they sometimes felt restricted as to the type of knowledge which they felt able to share.

## 4 DISCUSSION AND CONCLUSION

### 4.1 Discussion

Despite being a knowledge—rich environment, knowledge sharing is complex within health care and there are difficulties associated with it.^13^, ^14^ This paper has highlighted the influence of social and cultural aspects of the health‐care environment in this process.

Knowledge sharing is heavily influenced by the social roles of actors within health‐care interactions. Broadly, there are differences in expectations of what knowledge is shared and whether it is utilized between HCPs and patients. These differences are associated with the normative social roles of patient and HCP. These norms and associated behavioural expectations are very embedded within the health‐care context and largely viewed uncritically. This study has uncovered a more nuanced understanding of how these social roles influence knowledge sharing however. Specific professional roles appear to influence what knowledge is shared by patients. In addition, it would appear that particular knowledge is associated with the roles of patient and HCP. The suggestion is that this association of knowledge and role is also related to social norms, and becomes problematic if violated.

The contribution which this study makes is that these themes appear to be common for all patients regardless of characteristics such as age, gender, ethnicity, educational level or disease vintage, and likewise for HCPs. Current health literacy literature ^22^, ^23^, ^24^, ^25^ has often focused on educational or socio‐economic factors to explain patients' ability to contribute to health‐care decision making, but this study would dispute this being the only perspective on this issue. Likewise, the shared decision‐making literature has often focused on lack of effective communication skills or insufficient use of decision‐making models and strategies to explain difficulties in health‐care decision making.^18^, ^19^, ^20^, ^21^ By foregrounding knowledge itself, this study has shown that some elements of knowledge sharing required for health‐care decision making transcend the influences of socio‐economic factors and are contingent rather on sociocultural factors. It is not communication or decision‐making strategies which are required to move practice on, but a deeper understanding of the cultural context in which these interactions take place.

Whilst knowledge sharing may transcend some of the conventional influences such as education or communication skills often identified in the literature, it appears that different professional HCP roles do have an effect on how knowledge sharing is achieved. This appears to occur independently to some extent of any specific personal relationships between social actors. Nurses in particular were perceived to be more empathetic towards patients and therefore were used as a channel through which 'difficult' knowledge could be shared with medical staff. Again, this appears to be associated with the cultural attributes of each social role and the normative expectations of their attitudes and behaviours rather than the professional remit of their role as might be expected.

Asymmetry within doctor/patient interactions has been recognized previously,^39^ but this has often focused on communication skills and processes.^40^ One of the advantages of foregrounding knowledge itself is that its influence on how it is shared has been elucidated. It is clear that there are different types of knowledge, these types of knowledge are assigned differential value, and specific types of knowledge are associated with particular social roles. This is exemplified by higher value biomedical knowledge associated with HCPs in comparison with less valued experiential knowledge which patients contribute. The knowledge itself is therefore related to, and contributes to, the normative social roles of HCP and patient. Furthermore, violating these social norms in relation to knowledge can be problematic in health‐care interactions.

A deficit model of knowledge is common in health care which assumes that patients have insufficient knowledge in order to self‐manage.^41^ The goal of health care is therefore to furnish patients with the knowledge which they require to manage their conditions. In contrast, this study would suggest that it is not necessarily a lack of knowledge which is the issue, but rather an undervaluing of the unique experiential knowledge which patients have and a corresponding lack of opportunity to effectively utilize it within the decision‐making process.

Understanding the challenges of knowledge sharing is crucial to support multi‐directional knowledge sharing between patients and HCPs. This ensures that not only do patients have access to knowledge which allows them to self‐manage, but that they are able to feedback the success or otherwise of a programme of self‐management based on experiential knowledge. Only then can a personalized plan of care be developed which allows for individuals' health to be effectively managed within their unique set of circumstances.^17^ Most discussions regarding self‐management focus almost exclusively on 'increasing' the knowledge which patients have.^8^ Little consideration is given to whether patients feel able to utilize this knowledge within the routine interactions concerned with health‐care management and planning. Moreover, it has been highlighted that knowledge and skills, and the confidence to utilize them, decline over time unless supported in the longer term.^36^ Developing knowledge and skills and supporting in routine interactions the confidence to utilize them would be a cost‐effective approach, thereby removing the need for additional ongoing support mechanisms for patients.

The converse can, however, easily occur as a result of common practices within routine interactions. It has been identified in health care how patients can be perceived to be cognitively unreliable or emotionally compromised.^42^ This study has shown how this can manifest itself in everyday practices where patients' experiential knowledge is routinely perceived by HCPs and patients themselves, to be of lesser value than biomedical clinical knowledge. This in turn underpins the control which HCPs have over routine health care practices and the decision making which takes place within them. Without uncovering and acknowledging the incidences of this within routine behaviours, and seeking to change practice, patients will continue to find it challenging to participate in their own health‐care management in the way in which health‐care agendas purport to want them to.^43^

Multi‐disciplinary working, whilst beneficial in many ways, has been highlighted as being problematic in the arena of knowledge sharing.^14^ Differential knowledge sharing between patients and various members of the health‐care team can make it difficult for any one member to ascertain a comprehensive picture of the patient's self‐care activities and the impact of these. This work shows that possibilities for patients to share their unique knowledge with the health‐care team are increased by offering a range of interactions and relationships within which this can occur. This allows for more comprehensive knowledge sharing by patients when viewed from the perspective of the multi‐disciplinary team as a whole.

Whilst recent policy appears to endorse the value of patients' unique knowledge gained from lived experience, this is not always reflected in practice.^17^ Embedded practices of both HCPs and patients mean that the accepted value of different types of knowledge is often unchallenged thereby making it more difficult for sharing of patients' experiential knowledge due to its perceived lower value.

## 5 LIMITATIONS

This study has some limitations in its design. Comprehensive demographics including educational level, employment status and duration of illness were not collected on participants. As these characteristics can have a bearing on individuals' ability to participate in health‐care interactions, this information would have allowed additional elements of analysis to have been undertaken. This does not influence the findings in as much as the themes identified were common to all participants regardless of these or any other attributes, but the extent to which particular characteristics influenced practices was not able to be explored. In addition, the data were collected by a single researcher who had familiarity with the health‐care setting. Whilst this may have had advantages and was accounted for in the study design and data analysis, a larger study with more opportunity for inter‐researcher discussion may have strengthened the study.

### 5.1 Potential for further study

There are further avenues of research suggested by these results, one being to explore whether the issues identified are the same in primary care or general practice where HCPs are less specialist in particular areas of health‐care practice. Power dynamics within the relationships between HCP and patient may be different in this context for a variety of reasons, as well as the value of knowledge being shared within these interactions. In addition, there may be some value in exploring whether carers mediate knowledge sharing within health care. This was not possible in this study as there was only one participant who had carers present at their consultations, but exploring this further may be an interesting avenue of further study.

## 6 CONCLUSION

Knowledge sharing is a socially situated activity. Understanding the social context of knowledge sharing is crucial, as is understanding the roles and perspectives of the actors involved. As we try to encourage patients to take on more self‐management activities, it is essential to understand how this relates to their current understandings of their roles within health care and their relationships with other actors. It is not enough for policy to endorse the value of patients' unique knowledge unless there is support in practice to make this a reality.

Accepted social roles of both HCPs and patients are part of the culture of health care and exceptionally challenging to alter. Recognition of these roles—their benefits and challenges—along with an acknowledgement of practices which perpetuate them is the first step in addressing some of the issues which they present. Changes to practice can contribute to alteration of culture incrementally over time. This requires work with both HCPs and patients simultaneously as changes to the practices of one group will have little impact if met by a lack of understanding of on the part of other social actors.

The tension for patients in self‐management is that within the health‐care context of the hospital, their unique knowledge is undervalued, but once out of that environment, they rely on this to manage their health conditions. This is a paradox which requires addressing if patients are to feel empowered to manage their conditions based not only on knowledge and advice from HCPs, but also their own experiential knowledge. This could, and should, be reinforced through interactions in routine health‐care consultations.

## CONFLICT OF INTEREST

The authors have no conflicts of interest to declare.

## Data Availability

The data that support the findings of this study are available from the corresponding author upon reasonable request.

## References

[1] House of Commons Select Committee . Managing the Care of People With LTCs. Stationary Office; 2014.

[2] World Health Organisation . Preventing Chronic Diseases: a Vital Investment. World Health Organisation; 2005. www.who.int/chp/chronic_disease_report/en/ (accessed on 20.3.19)

[3] Schroeder SA . We can do better – improving the health of the American people. N Engl J Med. 2007;357:1221‐1228. 10.1056/NEJMsa073350 17881753

[4] US Department of Health and Human Service . Multiple chronic conditions—a strategic framework: Optimum Health and Quality of Life for Individuals With Multiple Chronic Conditions. Washington DC. US Department of Health and Human Services; 2010.

[5] NHS England . The NHS Belongs to The People: a Call to Action. Stationary Office; 2013.

[6] NHS England . Supporting self‐management/self care. https://www.england.nhs.uk/ourwork/patient‐participation/self‐care/ 2019 (accessed 20.3.19).

[7] Lorig KR , Sobel DS , Stewart AL , et al. Holman Evidence suggesting that a chronic disease self‐management program can improve health status while reducing hospitalization: a randomized trial. Med Care. 1999;37:5‐14. 10413387 10.1097/00005650-199901000-00003

[8] de Ionge A , Fagan P , Fenner J , Kidd L . A practical guide to self‐management support. Health Foundation; 2015.

[9] Bodenheimer T , Lorig K , Holman H , Grumbach K . Patient self‐management of chronic disease in primary care. J Amer Med Assoc. 2002;288:2469‐2475. 10.1001/jama.288.19.2469 12435261

[10] Hibbard JH , Stockard J , Mahoney ER , Tusler M . Development of the patient activation measure (PAM): conceptualising and measuring activation in patients and consumers. Health Serv Res. 2004;39(2004):1005‐1026. 10.1111/j.1475-6773.2004.00269.x 15230939 PMC1361049

[11] Hibbard JH , Gilburt H . Supporting People to Manage Their Health. An introduction to patient activation; 2014.

[12] Martin L , Williams S , Haskard K , DiMatteo M . The challenge of patient adherence. Ther Clin Risk Manag. 2005;1:189‐199. 18360559 PMC1661624

[13] Davenport TH , Beck JC . Just in time delivery comes to KM. Harvard Bus Rev. 2002;80:107‐111. 12140850

[14] Oborn E , Dawson S . Knowledge and practice is multidisciplinary teams: struggle, accommodation and privilege. Hum Relat. 2010;63:1835‐1857. 10.1177/0018726710371237

[15] Carlile PR . A pragmatic view of knowledge and boundaries: boundary objects in new product development. Organ Sci. 2002;13:529‐540.

[16] Carlile PR . Transferring, translating and transforming: An integrative framework for managing knowledge across boundaries. Organ. Sci. 2004;15:555‐568.

[17] Department of Health . Universal Personalised Care. Department of Health; 2019.

[18] Elwyn G , Frosch D , Thomson R , et al. Shared decision‐making: a model for clinical practice. Gen Intern Med. 2012;1361‐1367. 10.1007/s11606-012-2077-6 PMC3445676 22618581

[19] Barry MJ , Edgman‐Levitan S . Shared decision‐making – the pinnacle of patient‐centered care. N Engl J Med. 2012;780‐781. 22375967 10.1056/NEJMp1109283

[20] Gulbrandson P , Dalby A , Ofstad E , Gerwing J . Confusion in and about shared decision‐making in hospital outpatient encounters. Patient Educ Couns. 2014;96:287‐294. 25086447 10.1016/j.pec.2014.07.012

[21] Matthias MS , Salyers MP , Frankel RM . Frankel Re‐thinking shared decision‐making: context matters. Patient Educ Couns. 2013;91:176‐179. 23410979 10.1016/j.pec.2013.01.006

[22] Nutbeam D . Evolving concept of health literacy. Soc Sci Med. 2008;67:2071‐2078. 10.1016/j.socscimed.2008.09.050 18952344

[23] Berkman N , Davis T , McCormack L . Health literacy: what is it? J Health Commun. 2010;15:9‐19. 10.1080/10810730.2010.499985 20845189

[24] Brabers A , Rademakers J , Groenewegen P , Van Kijk L , de Jong JD What role does health literacy play in patients' involvement in medical decision making? PLoS One. 2017;12:e0173316. 28257472 10.1371/journal.pone.0173316 PMC5336280

[25] Nielson‐Bohlman C , Panzwer A , Kindig D (eds.) Health Literacy. A Prescription to End Confusion. The National Academies Press. 25009856

[26] Berkman N , Sheridan S , Donahue K , Halpern DJ , Crotty K . Low health literacy and health outcomes: an updated systematic review. Ann Intern Med. 2011;155:97‐107. 21768583 10.7326/0003-4819-155-2-201107190-00005

[27] Sandowska A , Soderlund J . Trust, reflexivity and knowledge integration: toward a conceptual framework concerning mobile engineers. Hum Relat. 2015;68:973‐1000.

[28] Romme A , Aenier M‐J , Denyer D , et al. Toward common ground and trading zones in management research and practice. Br J Manag. 2015;26:544‐559.

[29] Hsu J , Chu T , Lin T , Lo C . Coping knowledge boundaries between information system and business disciplines: an intellectual capital perspective. Inf Mang. 2014;51:283‐295.

[30] Alvesson M . Knowledge work: ambiguity, image and identity. Hum Relat. 2001;57(2001):863‐886.

[31] Cross R , Sproull L . More than an answer: Information relationships for actionable knowledge. Organ Sci. 2004;15:446‐462.

[32] Fetterman Ethnography DM . Step by Step, (3rd edn.). SAGE; 2010.

[33] Hammersley M , Atkinson P . Ethnography: principles in Practice. SAGE; 2007.

[34] Bray Z . Ethnographic approaches. In Della Porta D , Keating M (eds.) Approaches and Methodologies in the Social Sciences. A pluralist perspective. Cambridge Cambridge University Press; 2008.

[35] Dey I . Grounding categories. In: Bryant A , Charmaz K , eds. Handbook of Grounded Theory. SAGE; 2007:296‐315

[36] Charmaz K . Constructing Grounded Theory. SAGE, 2006.

[37] Gioia DA , Corley KG , Hamilton AL . Seeking qualitative rigor in inductive research: notes on the Gioia methodology. Organ Res Method. 2012;16:15‐31.

[38] Newbronner L , Chamberlain R , Borthwick R , Baxter M , Sanderson D . Sustaining and Spreading Self‐Management Support: lessons From Co‐Creating Health phase 2. The Health Foundation; 2013. http://www.health.org.uk/sites/default/files/SustainingAndSpreadingSelfManagementSupport.pdf

[39] Kenny A , Veldhuijzen W , van der Weijden T , et al. Interpersonal perception in the context of doctor‐patient relationships: a dyadic analysis of doctor‐patient communication. Soc Sci Med. 2010;70(5):763‐768. 20005618 10.1016/j.socscimed.2009.10.065

[40] Fricker M , Epistemic Injustice M . Power and The Ethics of Knowing. Oxford University Press; 2007.

[41] Zimbudzi E , Lo C , Misso M , Ranasinha S , Zoungas S . Effectiveness of management models for facilitating self‐management and patient outcomes in adults with diabetes and chronic kidney disease. Syst Rev. 2015;4:81. 10.1186/s13643-015-0072-9 26059649 PMC4489399

[42] Carel H , Kidd HJ . Epistemic injustice in healthcare: a philosophical analysis. Med Health Care Philos. 2014;17:529‐540. 10.1007/s11019-014-9560-2 24740808

[43] Plinick A , Dingwall R . On the remarkable persistence of asymmetry in doctor/patient interaction: a critical review. Soc Sci Med. 2011;72:1374‐1382. 21454003 10.1016/j.socscimed.2011.02.033

